# Heme as a Pro-Inflammatory Stimulus in Abdominal Aortic Aneurysm

**DOI:** 10.3390/antiox15020155

**Published:** 2026-01-23

**Authors:** Yuchao Ding, László Potor, Péter Sótonyi, Ágnes Szappanos, Gergő Péter Gyurok, Szilárd Póliska, Andreas Patsalos, Gábor Méhes, Lívia Beke, Katalin Éva Sikura, Erzsébet Zavaczki, Tamás Gáll, Dávid Pethő, Attila Fintha, Beáta Nagy, Béla Juhász, László Nagy, György Balla, József Balla

**Affiliations:** 1 Division of Nephrology, Department of Internal Medicine, Faculty of Medicine, University of Debrecen, 4032 Debrecen, Hungary; yuchao.ding@med.unideb.hu (Y.D.);; 2 HUN-REN-UD Vascular Pathophysiology Research Group, University of Debrecen, 4032 Debrecen, Hungary; 3 Kálmán Laki Doctoral School, University of Debrecen, 4032 Debrecen, Hungary; 4 Heart and Vascular Center, Department of Vascular Surgery and Endovascular Surgery, Semmelweis University, 1122 Budapest, Hungary; 5 Heart and Vascular Centre, Semmelweis University, 1122 Budapest, Hungary; 6 Department of Rheumatology and Clinical Immunology, Semmelweis University, 1122 Budapest, Hungary; 7 Department of Biochemistry and Molecular Biology, Faculty of Medicine, University of Debrecen, 4032 Debrecen, Hungary; 8 Department of Medicine, Division of Endocrinology, Diabetes & Metabolism, Johns Hopkins University School of Medicine, Institute for Fundamental Biomedical Research, All Children’s Hospital, St. Petersburg, FL 33701, USA; 9 Department of Pathology, Faculty of Medicine, University of Debrecen, 4032 Debrecen, Hungary; 10 Department of Pathology and Experimental Cancer Research, Semmelweis University, 1122 Budapest, Hungary; 11 Department of Pharmacology and Pharmacotherapy, Faculty of Medicine, University of Debrecen, 4032 Debrecen, Hungary; 12 Departments of Medicine and Physiology, Pharmacology and Therapeutics, and Biomedical Engineering, Johns Hopkins University School of Medicine, Institute for Fundamental Biomedical Research, All Children’s Hospital, St. Petersburg, FL 33701, USA; 13 Department of Pediatrics, Faculty of Medicine, University of Debrecen, 4032 Debrecen, Hungary

**Keywords:** abdominal aortic aneurysm, methemoglobin, heme, heme oxygenase-1, vascular inflammation

## Abstract

Abdominal aortic aneurysm (AAA) is a lethal vascular disease characterized by intramural hemorrhage. This study delineates the signatures of heme and its metabolic imbalance related to progression and inflammation in AAA. Clinical analyses of patients undergoing open AAA surgery show that AAA patients exhibit vascular inflammation, with elevated serum CRP, IL-6, and heme levels correlating with the expression of heme-regulated gene *Hmox1/HO-1* (heme oxygenase-1) in the affected aortic wall. Oxidation of hemoglobin to ferri state leading to accumulation of methemoglobin readily releasing heme occurs in human AAA and in angiotensin II (AngII)-induced AAA in apolipoprotein E-deficient mice. Transcriptomic analysis for AngII-induced AAA identifies upregulated genes predominantly enriched in inflammatory signaling, extracellular matrix degradation, oxidative stress pathways, and altered expression of genes related to heme metabolism including *Hmox1*. Immunohistochemistry for IL1β and TNFα confirms inflammatory activation within AAA tissues. The signatures of heme-responsive gene inductions, enhanced expression of HO-1 and H-ferritin, are detected. Mechanistic studies employing endothelial cells and smooth muscle cells reveal that heme exposure of resident cells markedly enhances the expression of *IL1β* and *ICAM1*, as well as the inflammasome component *NLRP3*, and such inflammatory response is controlled by HO-1. Intervention with Normosang (heme arginate), an HO-1 inducer, attenuates aneurysm progression, whereas HO-1 inhibition by Tin protoporphyrin IX abolishes this protection. Induction of HO-1 accompanied by elevated H-ferritin level also mitigated aortic wall inflammation as reflected by lowering IL1β and TNFα. These findings highlight the heme-HO-1-H-ferritin axis as an element of AAA pathogenesis and a potential therapeutic target.

## 1. Introduction

Abdominal aortic aneurysm (AAA) is a progressive and potentially fatal vascular disorder characterized by localized dilation of the aorta, extracellular matrix degradation, inflammatory cell infiltration, and risk of rupture [1,2,3,4,5]. Despite advances in understanding AAA pathophysiology, therapeutic options remain limited, and the mechanisms driving aneurysm progression are incompletely defined.

Accumulating indirect observations suggest that heme oxygenase-1 (HO-1) activity is protective in AAA [6,7]. HO-1 deficiency accelerates aneurysm formation and rupture in mice, and genetic variants associated with lower HO-1 inducibility correlate with increased AAA prevalence in humans [8]. Intramural hemorrhage of arterial wall represents a potential hemoglobin (Hb) and heme burden in AAA. Auto-oxidation of Hb is a continuous reaction that leads to methemoglobin (metHb) generation and concomitant formation of superoxide anions. Neutrophil granulocyte- and macrophage-derived reactive oxygen species and oxidized lipids of atherosclerotic lesion greatly amplify Hb oxidation and the formation of metHb [9,10], known to readily release its heme moieties [11]. However, it remains unclear whether heme toxicity driven by Hb oxidation constitutes a primary causal driver of aneurysmal progression, whether metHb accumulation is a conserved feature across species, and whether pharmacological augmentation of HO-1 activity can therapeutically restrain AAA evolution remains unresolved.

Heme acts both as a substrate and inducer of HO-1 via transcription factor Bach1 [12,13,14]. Normosang (heme arginate) used in the treatment of acute hepatic porphyria [15] is an effective HO-1 gene regulator, but unlike heme, it does not amplify oxidative cellular damage [16]. Given that Normosang is a clinically approved HO-1 inducer that does not exacerbate oxidative stress, it is an ideal candidate to augment HO-1 activity and assess its therapeutic potential in AAA.

Healthy endothelial cells (ECs) and smooth muscle cells (SMCs) are essential for maintaining vascular homeostasis, regulating vascular tone, and preserving an anti-inflammatory and antithrombotic environment within the vessel wall. While ECs play a key role in barrier function and inflammatory regulation, SMCs are critical for maintaining structural integrity and extracellular matrix homeostasis of the arterial wall. Growing evidence indicates that disruption of endothelial function represents an early pathogenic event in AAA development, which in turn promotes inflammatory processes, phenotypic switching, and loss of SMCs, thereby contributing to progressive arterial wall degeneration [17,18].

In this study, we identify metHb-derived heme overload as a central pathogenic mechanism in AAA. We demonstrate that aneurysmal tissues in both mice and humans are characterized by marked accumulation of oxidized Hb species, elevated free heme burden, and a conserved iron-loading, pro-oxidant transcriptional signature with robust induction of HO-1. Mechanistic studies on ECs and SMCs further reveal that heme exposure of resident cells markedly enhances the expression of interleukin-1 beta (*IL1β*), intercellular adhesion molecule 1 (*ICAM1*), as well as the inflammasome component NOD-, LRR-, and pyrin domain-containing protein 3 (*NLRP3*), and such inflammatory response is controlled by HO-1. We further show that pharmacological HO-1 induction with Normosang markedly attenuates aneurysm formation and intramural hemorrhage, whereas HO-1 inhibition aggravates disease severity. These findings establish the heme-HO-1 axis as a modifiable upstream driver of AAA pathogenesis and reveal therapeutic potential for targeting heme stress to halt aneurysmal progression.

## 2. Materials and Methods

### 2.1. Human Samples

Surgical intervention was indicated for infrarenal AAA with diameters ≥ 50 mm in women and ≥55 mm in men without rapid progression or symptoms. Open repair was selected for patients with low to moderate surgical risk or when aneurysm morphology was unsuitable for endovascular repair. Abdominal ultrasound and computed tomography angiography (CTA) were used for diagnosis and prognostic evaluation, and preoperative CTA was performed in all patients. Adult patients scheduled for open AAA repair were eligible for inclusion. Written informed consent was obtained from all participants. Exclusion criteria included age < 18 or > 80 years, inability to comply with study procedures, or pregnancy.

Samples taken from the vessel walls of 67 patients during open aortic aneurysm surgery underwent detailed histological analysis. Two independent expert cardiovascular pathologists (A.F. and B.N.) performed the histological analysis of the specimens. The histological analysis required to determine the inflammation of the vessel wall in a 3-tier score (absence of inflammation, subclinical inflammation, well-defined inflammation) is carried out according to a histopathological classification scheme published by Bruijn LE et al. [19].

Serum C-reactive protein (CRP) levels were measured and expressed in mg/L; values exceeding 5 mg/L were considered elevated. CRP levels were measured using an immunoturbidimetric detection method on a Beckman Coulter DxC 700 AU chemistry analyzer. Serum interleukin-6 (IL-6) concentrations (pg/mL) were determined using an electrochemiluminescence immunoassay performed on a Cobas E601 analyzer (Roche Diagnostics, Indianapolis, IN, USA), with levels above 7 pg/mL considered indicative of elevated inflammation. In the patient cohort examined, CRP and IL-6 levels showed no association with several key comorbid conditions (hypertension, diabetes mellitus, hyperlipidemia, or smoking).

RNA-seq data generated in this study are publicly accessible through the NCBI BioProject database (accession no. PRJNA1196652) [20].

### 2.2. Abdominal Aortic Aneurysm Induced by Subcutaneous Angiotensin II Infusion Using Osmotic Pumps in Apolipoprotein E-Deficient Mice

Apolipoprotein E-deficient (ApoE^−/−^) mice on a C57BL/6J background, aged 8–10 weeks (47 males and 10 females; body weight 25 ± 5 g) were provided by the Department of Laboratory Animal Science, University of Debrecen, and kept under specific pathogen-free conditions following the approval and guidelines of the Institutional Ethical Committee. Animal experiments were performed in compliance with the guidelines of Directive 2010/63/EU of the European Parliament concerning the protection of animals used for scientific research.

**Figure 1:**
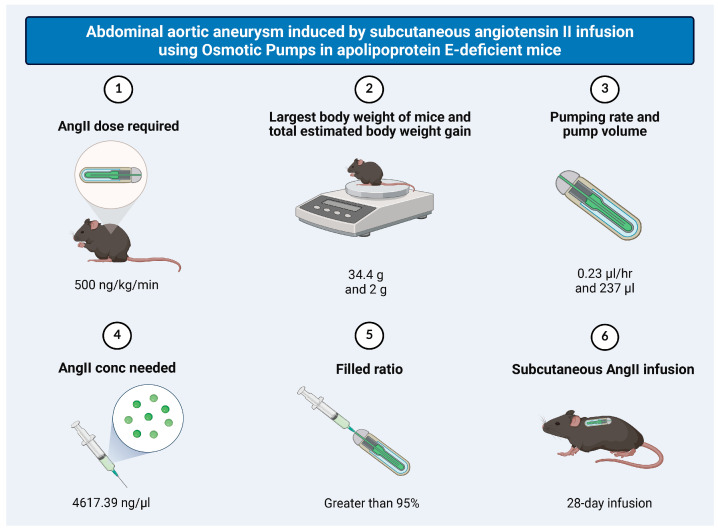
Abdominal aortic aneurysm induced by subcutaneous angiotensin II infusion using Osmotic Pumps in apolipoprotein E-deficient mice. AngII infusion was performed using osmotic minipumps as follows. ApoE^−/−^ mice were weighed prior to pump preparation to calculate the required AngII dose. Based on previous experience, a projected body weight gain of 2 g over the 4-week infusion period was included in the calculation. AngII was administered at a dose of 500 ng/kg/min for 4 weeks. The AngII concentration was determined using the “mean pumping rate” specified by the manufacturer’s pump instructions. Each osmotic pump was filled with approximately 237 μL of AngII solution. To ensure accurate dosing, pumps were weighed before and after filling, and the filling rate was calculated. Only pumps with a filling rate greater than 95% were used for implantation.

Mice were fed a high-fat diet (HFD) containing 18% protein, 12.6% carbohydrate, 15% fat, and 1.25% cholesterol (Sniff Spezialdiäten GmbH, DE-59494, Soest, Germany) for 12 weeks. During the last four weeks, angiotensin II (AngII) (Sigma-Aldrich Corp., St. Louis, MO, USA) was continuously administered subcutaneously at 500 ng/min/kg using an osmotic minipump (Model 2004; ALZA Scientific Products, Mountain View, CA, USA) (Figure 1).

The experimental mice were first anesthetized and fixed on the operating table. Mice were disinfected with iodine and alcohol after depilating their necks. An approximately 1 cm incision was made in the neck using surgical scissors, after which surgical tweezers were gently inserted to bluntly dissect the tissue and create a subcutaneous pocket. The Osmotic Pumps equipped with AngII were immersed in saline at a temperature of 37 °C for 24 h in advance. The top of the pump extends into the created pocket towards the tail of the mouse. The wound was closed with surgical sutures and disinfected again with iodine and alcohol.

At the end of the study period, mice were euthanized by carbon dioxide asphyxiation. Each mouse’s aorta was dissected and rinsed with ice-cold saline, and the adjacent periaortic tissue was excised. The aorta was soaked in cold saline, the connective tissue around the aorta under a stereomicroscope was removed, and the aorta was separated. The arteries were photographed at the macroscopic level using a Nikon D3200 camera (Nikon Corp.; Minato, Tokyo, Japan).

For Normosang and Tin protoporphyrin IX (SnPP) treatment, mice received intraperitoneal injections of 18 mg/kg Normosang (25 mg/mL, concentrate for solution for infusion, Recordati Rare Diseases UK Ltd., N2210-01, London, England, UK) and 10 mg/kg SnPP (CAS Number: 14325-05-4, Sigma-Aldrich Corp., St. Louis, MO, USA) every other day starting from week 0 and continuing until the end of the 12-week study period. Four experimental groups were included, with 10 mice per group: Control, AngII+HFD (AAA), AngII+HFD+Normosang+SnPP, and AngII+HFD+Normosang.

### 2.3. Abdominal Ultrasonography in Mice

The abdominal aorta of each mouse was imaged in vivo using the Vevo 3100 Imaging Station equipped with an MX550D linear transducer (FUJIFILM VisualSonics, Inc., Toronto, ON, Canada). Two-dimensional scans were conducted at 40 MHz, while Color Doppler measurements were obtained at 32 MHz. Mice were anesthetized with ketamine/xylazine (100/10 mg/kg, i.m.), and the abdominal hair was removed. The animals were then placed supine on the imaging platform, and ultrasound gel was applied to the skin. Long-axis images of the suprarenal aorta were acquired from the aortic hiatus to the left renal artery, with capture performed at mid-systole.

### 2.4. Western Blot

Healthy murine aorta and AAA tissues were cut into 10 mm pieces and homogenized in ice-cold protein lysis buffer containing protease inhibitor cocktail. Samples were sonicated three times for 10 s on ice and centrifuged at 12,000× *g* for 15 min at 4 °C. The total protein concentration of each lysate was determined using a BCA protein assay kit (Thermo Fisher Scientific, Waltham, MA, USA) according to the manufacturer’s instructions. Equal amounts of protein (10 µg per sample) were mixed with 4× Laemmli sample buffer, boiled for 5 min, and loaded onto 10% SDS-polyacrylamide gels. After electrophoresis, proteins were transferred onto nitrocellulose membranes (Amersham Biosciences, Piscataway, NJ, USA). Membranes were blocked for 1 h at room temperature in 5% non-fat dry milk in TBS-Tween (TBST) and then incubated overnight at 4 °C with primary antibodies: rabbit anti-mouse HO-1 (cross-reactive for both human and mouse; 10701-1-AP, dilution: 1:5000; Proteintech, Rosemont, IL, USA), rabbit anti-mouse ferritin heavy chain (H-ferritin; cross-reactive for both human and mouse; sc-376594, dilution: 1:500; Santa Cruz Biotechnology, Dallas, TX, USA). After washing with TBST (3 × 10 min), membranes were incubated with HRP-conjugated secondary antibodies for 1 h at room temperature. Protein bands were visualized using enhanced chemiluminescence (GE Healthcare, Piscataway, NJ, USA) and captured using a digital imaging system.

### 2.5. Analysis of Various Redox States of Hemoglobin

Healthy murine arteries, murine AAA tissues, and human healthy aorta and AAA samples (n = 7/group) were placed in saline and homogenized by sonication (three cycles of 10 s on ice) using an Ultrasonic Cell Disrupter (VirTis, Model 323410, Gardiner, NY, USA). The homogenates were centrifuged at 12,000× *g* for 15 min at 4 °C, and the supernatants were subjected to spectrophotometric analysis (Beckman Coulter Inc., Brea, CA, USA).

Absorbance values were recorded at 560 nm, 576 nm, and 630 nm, corresponding to the characteristic wavelengths of hemoglobin redox species. The concentrations of oxyhemoglobin (oxyHb) and metHb were calculated using previously established equations by Alayas [21]:

[OxyHb] = −75.78OD_560_ + 103.16OD_576_ − 38.39OD_630_

[MetHb] = −26.09OD_560_ + 12.48OD_576_ − 280.70OD_630_

### 2.6. Determination of Total Heme Content

Heme measurements in the healthy aorta and AAA tissue samples were performed as previously described by our study and by Huy et al. [20,22]. Briefly, tissues were homogenized in Tris-HCl buffer (SERVA Electrophoresis GmbH, 201391, Heidelberg, Germany), mixed with 2 M oxalic acid, and heated at 95 °C for 30 min to convert heme into protoporphyrin IX. Fluorescence (Ex 405 nm/Em 600 nm) was measured and heme concentrations were calculated from a hemin standard curve and normalized to protein content.

### 2.7. Histology

Tissue sections (4 µm) from representative formalin-fixed paraffin-embedded aortic specimens were processed for serial hematoxylin-eosin (H&E), Verhoeff-Van Gieson (EVG), Masson’s trichrome, and immunohistochemistry (IHC). After deparaffinization and rehydration, antigen retrieval was performed in citrate buffer (10 mM, pH 6.0) using microwave heating for 20 min at 95 °C. Endogenous peroxidase activity was quenched with 3% H_2_O_2_ for 10 min, and nonspecific binding was blocked with 5% normal goat serum (or 1% BSA) for 30 min at room temperature.

Primary antibodies were applied as follows: anti-HO-1 (Proteintech, 10701-1-AP, Rosemont, IL, USA) at 1:400 for 1 h at room temperature; anti-H-ferritin (rabbit monoclonal, clone EPR3005Y; Abcam, ab75972, Cambridge, UK) at 1:50 for 1 h at room temperature; anti-IL1β (polyclonal; Thermo Fisher Scientific, P420B, Rabbit IgG, Waltham, MA, USA) at 1:100 for 1 h at room temperature; and anti-TNFα (polyclonal; Thermo Fisher Scientific, PA5-19810, Waltham, MA, USA) at 1:100 for 1 h at room temperature. After three PBS washes, slides were incubated with MACH2 Rabbit HRP-Polymer (Biocare Medical, RP520H, Pacheco, CA, USA) for 30 min at room temperature according to the manufacturer’s instructions. Chromogenic detection was performed with DAB and development was monitored microscopically. Slides were counterstained with Mayer’s hematoxylin, rinsed in running tap water for 10 min, dehydrated through graded alcohols, cleared in xylene, and mounted. For digital documentation, slides were scanned with a Mirax Midi scanner (3D Histech, Budapest, Hungary).

Quantitative evaluation of HO-1, H-ferritin, IL1β, and TNFα immunostaining was performed using ImageJ (1.53k, NIH, Bethesda, MD, USA). Images were converted to 8-bit grayscale, and a consistent threshold was applied to isolate DAB-positive (reddish-brown) staining.

### 2.8. Cell Culture

Human aortic endothelial cells (HAoECs) and human aortic smooth muscle cells (HAoSMCs) were purchased from Lonza (Lonza Group Ltd., Allendale, NJ, USA). HAoECs were cultured in CM199, while HAoSMCs were maintained in Dulbecco’s Modified Eagle’s Medium, both supplemented with 10% fetal bovine serum (FBS), 100 U/mL penicillin, 100 µg/mL streptomycin, and amphotericin B. Cells were expanded to approximately 90% confluence and used between passages 4 and 6, with media refreshed every 48 h. Heme treatments were performed in serum- and antibiotic-free medium as previously described [23]. In brief, hemin chloride stock solution prepared in sterile 20 mM NaOH was diluted in the serum- and antibiotic-free medium. Cells were washed twice with Hank’s Balanced Salt Solution (pH 7.4) containing Ca^2+^ and Mg^2+^ (HBSS+) and exposed to varying concentrations of hemin for 2 h. After treatment, cells were washed with HBSS+, fresh medium containing 10% FBS and antibiotics was added, and cells were incubated for an additional 3, 6, or 16 h in a 5% CO_2_ incubator.

### 2.9. siRNA Transfection

Silencer select small interfering RNA (siRNA) targeting *HO-1* (Thermo Fisher Scientific, 4390824, Waltham, MA, USA) and a corresponding negative control siRNA were purchased from Ambion (Thermo Fisher Scientific, 4390843, Waltham, MA, USA). HAoECs transfection with siRNA was achieved as described earlier [23] using the Oligofectamine (Invitrogen, 12252011, Carlsbad, CA, USA) according to the manufacturer’s guide using 10 nmol/L siRNAs.

### 2.10. RNA Isolation and Quantitative Reverse Transcription-Polymerase Chain Reaction (qRT-PCR)

Total RNA was isolated from cells grown in six-well plates using TriReagent (Zymo Research, Cat. No. R2050-1-200, Irvine, CA, USA) according to the manufacturer’s instructions. cDNA synthesis was performed with the High-Capacity cDNA Reverse Transcription Kit (Applied Biosystems Inc., Cat. No. 4368813, Foster City, CA, USA). Quantitative PCR analysis of *HO-1*, *IL1β*, *ICAM1*, and *NLRP3* transcripts was carried out using TaqMan Gene Expression Assays (Thermo Fisher Scientific, Cat. No. EP0405, Waltham, MA, USA) with *GAPDH* as the endogenous control (HO-1: Hs01110250; IL1β: Hs01555410_m1; ICAM1: Hs00164932_m1; NLRP3: Hs00918082; GAPDH: Hs02758991_g1). Reactions were run on a C1000 Thermal Cycler coupled with the CFX 96 Real-Time PCR System (Bio-Rad, Serial No. 785BR03880, Hercules, CA, USA), and relative gene expression was calculated using the ΔΔCt method.

### 2.11. RNA-Sequencing

Total RNA was isolated from both murine and human aneurysm tissues. RNA quantity and integrity were evaluated using an Agilent BioAnalyzer together with the Eukaryotic Total RNA Nano Kit (Agilent Technologies, Santa Clara, CA, USA), following the manufacturer’s guidelines. Sequencing libraries were generated for analysis on an Illumina sequencing platform (Illumina, San Diego, CA, USA). Because the RNA obtained from aneurysmal tissue exhibited substantial degradation, we used an rRNA removal strategy. Ribosomal RNA was depleted using the NEBNext^®^ rRNA Depletion Kit (Human/Mouse/Rat) (New England BioLabs).

For library construction, 200 ng of total RNA was processed with the NEBNext^®^ Ultra II RNA Library Preparation Kit for Illumina (New England BioLabs, Ipswich, MA, USA). In brief, poly(A)+ transcripts were enriched using oligo-dT magnetic beads, after which the captured RNA was heat-fragmented at 94 °C for 15 min. First-strand cDNA synthesis was performed with random primers, followed by second-strand synthesis to yield double-stranded cDNA. After end repair and adaptor ligation, the resulting fragments were enriched through limited-cycle PCR to produce sequencing-ready libraries. Sequencing was carried out on an Illumina NextSeq 500 using a single-end, 75-cycle format.

The FASTQ reads were aligned to the mouse reference genome (GRCh37) using HISAT2, and the resulting BAM files were further analyzed with iDEP 2.01, http://ge-lab.org/idep/(accessed on 16 January 2024). Read counts were normalized with the DESeq2 algorithm, which was also used to identify differentially expressed genes between healthy aorta and aneurysmal tissue. Visualizations including heatmaps and dot plots were generated either within iDEP 2.01 or using GraphPad Prism 10 (GraphPad Software Inc., Version 10.1.1, La Jolla, CA, USA).

### 2.12. Statistical

All statistical procedures were performed using GraphPad Prism version 10 (GraphPad Software Inc., Version 10.1.1, La Jolla, CA, USA). Quantitative data are expressed as the mean ± standard error of the mean (SEM). Normal distribution of the datasets was verified with the Shapiro–Wilk test, and variance equality was examined using Levene’s test. When both assumptions were met, comparisons between two groups were carried out using the unpaired Student’s *t*-test, while analyses involving more than two groups were evaluated with one-way ANOVA followed by Bonferroni’s post hoc correction. For multi-factor experiments, two-way ANOVA followed by Sidak’s multiple comparisons test was used, as specified in the corresponding figure legends. Correlation analyses were performed using Pearson’s correlation coefficient. A *p* value < 0.05 was considered statistically significant.

## 3. Results

### 3.1. Inflammation-Linked to Heme Imbalance in Human Abdominal Aortic Aneurysm

**Figure 2:**
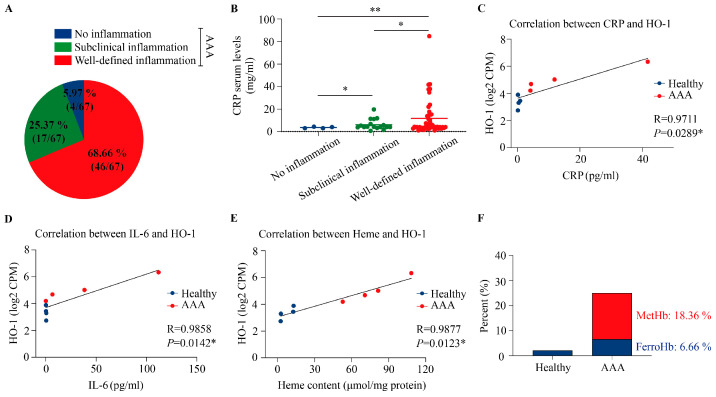
Inflammatory stratification and heme burden in human abdominal aortic aneurysm. (**A**), Representative distribution of inflammatory patterns in human AAA wall specimens. Red indicates well-defined inflammation (68.66%, n = 46), green indicates subclinical inflammation (25.37%, n = 17), and blue indicates absence of inflammation (5.97%, n = 4). (**B**), Serum CRP levels in patients stratified by inflammatory status. Data are presented as mean ± SEM. * *p* < 0.05, ** *p* < 0.01 (unpaired *t*-test). The correlation between CRP level and *HO-1* mRNA level (**C**), the correlation between IL-6 and *HO-1* mRNA level (**D**), and the correlation between Heme and *HO-1* mRNA level (**E**) of the aortic wall from human assessed using Pearson’s correlation analysis. Correlation coefficient (R) and *p* values were calculated. A two-tailed * *p* < 0.05 was considered statistically significant. (**F**), The presence of different Hb redox states (FerroHb and MetHb) was calculated as a percentage of the total heme content.

Given the hemorrhagic and oxidative milieu characteristic of human AAA, we stratified patients according to the degree of vascular inflammation. A total of 67 patients who underwent open AAA repair were included in the analysis. The mean age was 68.9 ± 6.8 years, and the male-to-female ratio was 52:15. Histopathological evaluation revealed well-defined inflammation in 68.7% of patients (n = 46) and subclinical inflammation in 25.4% (n = 17), accounting for 94.0% of all cases. Only 5.9% (n = 4) showed no evidence of vessel wall inflammation (Figure 2A). We next measured serum CRP levels in these patients. CRP concentrations correlated with the degree of inflammation, averaging 11.80 ± 15.89 mg/L in the well-defined inflammation group, 6.22 ± 4.76 mg/L in the subclinical group, and 3.60 ± 0.71 mg/L in patients without inflammation (Figure 2B). Correlation analyses revealed significant positive associations between CRP level and *HO-1* mRNA level in AAA tissue (Figure 2C), between IL-6 and *HO-1* mRNA level in AAA tissue (Figure 2D), and between heme and *HO-1* mRNA level in AAA tissue (Figure 2E) linking local inflammatory activation with the induction of HO-1 within the aneurysmal wall, known to be regulated by heme.

Since oxidative milieu in hemorrhagic lesion leads to oxidation of Hb and globin was shown to readily release its heme moiety after one electron oxidation to metHb [10], we performed spectrophotometric analysis from human aneurysmal aortas. Spectrophotometric profiling revealed absorbance signatures consistent with ferroHb (6.66%) and metHb (18.36%) in AAA, with oxidized Hb species comprising a substantially greater fraction of total heme content compared with healthy aortas, confirming an increased Hb oxidation burden in human AAA (Figure 2F).

### 3.2. Male Mice Exhibit Higher Susceptibility to Abdominal Aortic Aneurysm Formation

**Figure 3:**
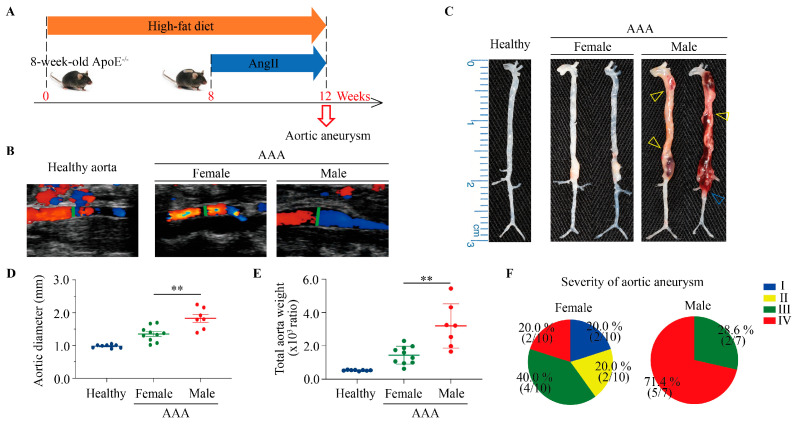
Male mice show a higher susceptibility to abdominal aortic aneurysm compared with female mice. (**A**), Experimental Schema: 8-week-old ApoE^−/−^ male or female mice were maintained on an HFD for a total duration of 12 weeks. During the last 4 weeks of feeding, they were implanted with osmotic pumps containing AngII. (**B**), Representative high-frequency ultrasound frames of aortas from healthy, female AAA, and male AAA groups. (**C**), Representative images of aortas: healthy aorta (**left**), female AAA (**middle**), and male AAA (**right**). Yellow triangles indicate an aortic aneurysm with hemorrhage; blue triangles indicate a ruptured aorta. Quantification of aortic diameter (**D**) and overall aortic weight (**E**) in healthy and AAA mice. Data are presented as mean ± SEM, n  =  7–10. ** *p* < 0.01 (unpaired *t*-test). (**F**), Severity grading of aortic aneurysm, categorized into four grades: Stage I, normal aorta; Stage II, aortic aneurysm without hemorrhage; Stage III, aortic aneurysm with hemorrhage; and Stage IV, ruptured aorta.

In order to study the signatures of heme overload in an animal model that resembles human pathology we employed AngII-induced AAA in ApoE^−/−^ mice. Since significant gender difference exists in the prevalence of AAA in humans [24] we investigated sex-related differences in aortic aneurysm susceptibility in AngII-induced AAA in ApoE^−/−^ mice. Eight-week-old male and female ApoE^−/−^ mice were fed an HFD for twelve weeks, with AngII infusion during the final four weeks (Figure 3A). Ultrasound imaging (Figure 3B) and gross morphological examination (Figure 3C) of the aorta revealed that male mice developed markedly larger aortic dilations and more extensive aneurysmal lesions compared with female mice. Quantitative analysis demonstrated that both the aortic diameter and total aortic weight were markedly higher in male AAA mice (Figure 3D,E; ** *p* < 0.01). Furthermore, severity of AAA indicated a higher proportion of severe (Stage III-IV) aneurysms in males (Figure 3F), suggesting that male sex confers greater vulnerability to AngII-induced AAA development.

### 3.3. Methemoglobin and Heme Accumulations Within Aortic Wall Are Characteristics of Abdominal Aortic Aneurysm in Male Mice

**Figure 4:**
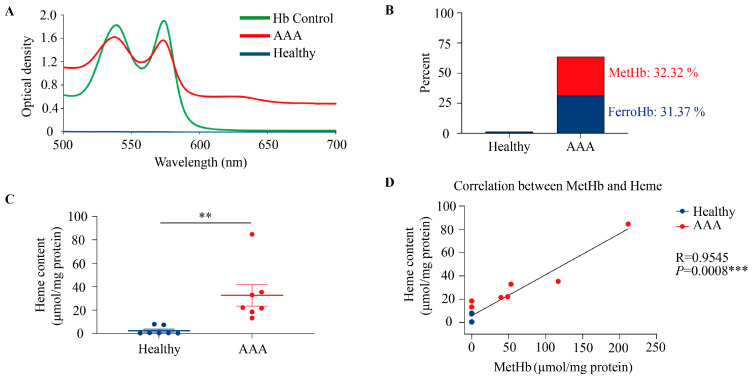
Methemoglobin and heme exposure in abdominal aortic aneurysm in mice. (**A**), Spectral analysis of aortic tissue from healthy aorta and AAA (n  =  7). The concentrations of various redox states of Hb were determined. (**B**), The presence of different Hb redox states was calculated as a percentage of the total heme content. (**C**), Total heme was measured by the fluorescence assay (n  =  7). The results are shown as the mean values  ±  SEM of the experiments. ** *p* < 0.01 (unpaired *t*-test). (**D**), The correlation between metHb and heme content of the aortic wall from mice was assessed using Pearson’s correlation analysis. Correlation coefficient (R) and *p* values were calculated. *** *p* < 0.001 (two-tailed).

Given the hemorrhagic nature of advanced AAA, spectrophotometric analysis of aortic tissues revealed distinct absorbance patterns characteristic of ferroHb and metHb in AAA samples derived from male ApoE^−/−^ mice on HFD for 12 weeks, with AngII infusion during the final 4 weeks (Figure 4A). Quantification of the different Hb redox states as a percentage of total heme content confirmed that oxidized Hb species were significantly enriched in aneurysmal tissues compared to controls (Figure 4B). Consistently, total heme levels, measured using a fluorescence assay, were substantially elevated in AAA samples (Figure 4C; ** p < 0.01), reflecting heme release from oxidized Hb within the vessel wall. Finally, correlation analysis between metHb and total heme content across human and mouse AAA samples revealed a strong positive association (Figure 4D; Pearson’s R = 0.9545, *p* = 0.0008), supporting the link between Hb oxidation and heme accumulation in hemorrhagic aneurysms.

### 3.4. Transcriptomic Profiling Identifies Distinct Molecular Signatures in Hemorrhagic Abdominal Aortic Aneurysm in Mice

**Figure 5:**
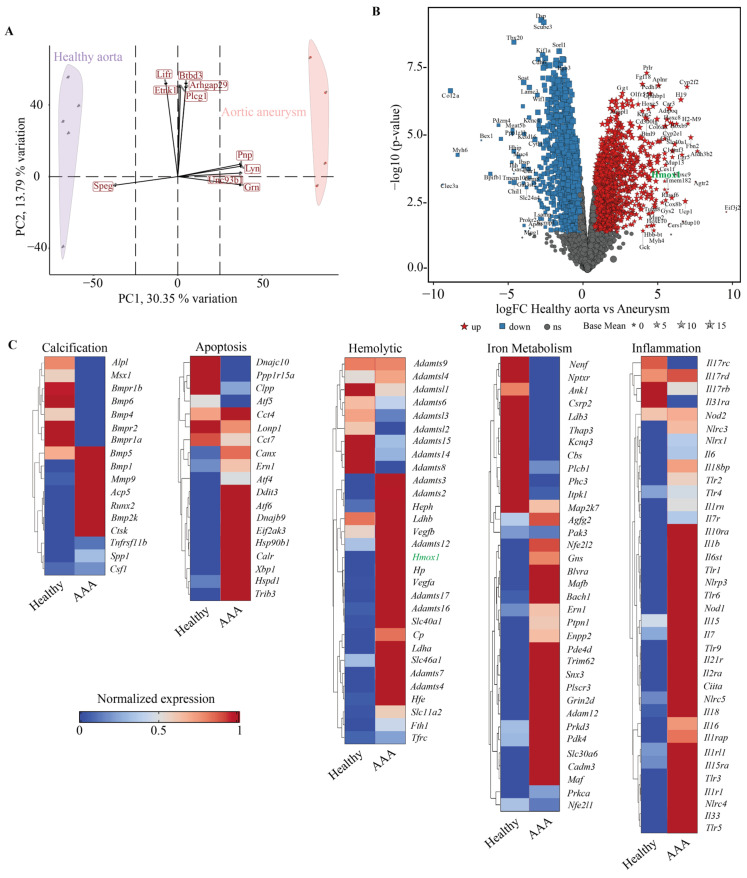
Transcriptomic differences between healthy aortic tissue and hemorrhaged aortic aneurysm in mice. (**A**), RNA sequencing analysis was performed on aortic samples from healthy mice (n = 5, purple circles) and mice with aortic aneurysm (n = 4, red circles). PCA illustrates the separation between the two groups. Unit variance scaling and singular value decomposition with imputation were applied to calculate principal components. The X- and Y-axes represent principal component 1 (PC1, 30.35% variance) and principal component 2 (PC2, 13.79% variance), respectively. (**B**), Differentially expressed genes (DEGs) are shown in a volcano plot, with red dots indicating upregulated genes, blue dots representing downregulated genes, and gray dots denoting genes without significant differential expression. (**C**), Clustered heatmap analysis illustrates the absolute expression profiles of genes related to Calcification, Apoptosis, Hemolysis, Iron Metabolism, and Inflammation in healthy versus AAA groups.

To gain mechanistic insights into the molecular pathways associated with AAA formation, RNA sequencing (RNA-seq) was performed on aortic tissues from healthy and aneurysmal male mice. Principal component analysis (PCA) demonstrated clear separation between the two groups, indicating substantial transcriptomic divergence (Figure 5A). Differential gene expression analysis identified numerous significantly upregulated and downregulated genes in AAA tissue (Figure 5B). The upregulated genes were predominantly enriched in inflammatory signaling, extracellular matrix degradation, and oxidative stress pathways, suggesting heme-driven vascular injury and remodeling.

Hierarchical clustering of differentially expressed genes revealed coordinated transcriptional changes across key biological processes (Figure 5C). AAA tissues showed increased expression of genes related to hemolysis and iron metabolism, including *Hmox1*, *Fth1*, and *Slc40a1*, indicating an active heme-catabolic response. Enhanced inflammatory gene expression (e.g., *IL1β*, *IL16*, *IL7*) and apoptotic markers further underscored the pro-inflammatory and degenerative environment of AAA. These findings highlight a multifaceted molecular remodeling process driven by Hb-heme exposure. The inverse expression of *Hmox1* and the iron-related genes including *Fth1* (Table 1), namely the induction of *Hmox1* and the lower expression of *Fth1* in the affected aortic wall compared to healthy samples reflects that iron metabolism is regulated mainly at translational level.

### 3.5. Histopathological Alterations and Heme-Related Protein Expression in Abdominal Aortic Aneurysms in Mice

**Figure 6:**
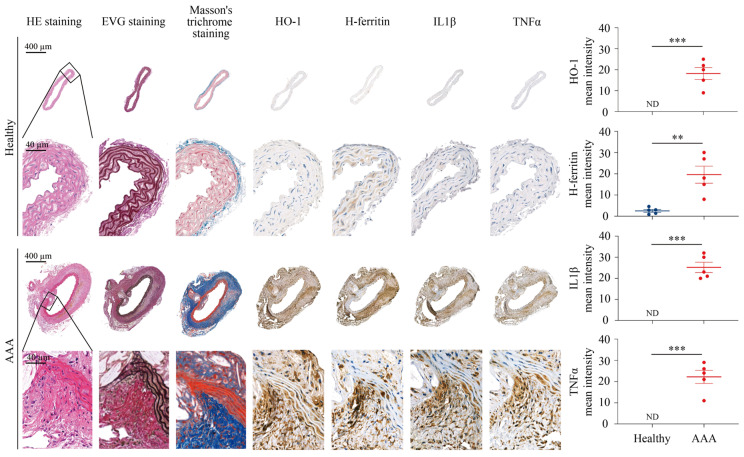
Histopathological analysis of healthy aortic tissue and abdominal aortic aneurysm in mice. Representative healthy aortic tissue sections and AAA tissue sections were examined using histological staining and IHC. H&E staining illustrates overall tissue morphology, EVG staining highlights elastic fibers, and Masson’s trichrome staining visualizes collagen and connective tissue. HO-1, H-ferritin, IL1β, and TNFα staining was performed to assess protein expression. Lower panels present magnified views of the corresponding regions. Quantitative analysis of HO-1, H-ferritin, IL1β, and TNFα staining of tissue sections was performed using ImageJ software (n = 5). ** *p* < 0.01, *** *p* < 0.001 (unpaired *t*-test). ND, not detectable. Mean intensity refers to the mean gray value of the DAB-positive signal within the defined region of interest. The units are expressed as arbitrary units (a.u.), which is standard for ImageJ-based densitometric quantification. Effect size analysis showed large to extremely large effects for all markers (HO-1 effect size = 4.08, mean difference = 18.2; H-ferritin effect size = 2.65, mean difference = 17.053; IL1β effect size = 4.32, mean difference = 25.2; TNFα effect size = 4.34, mean difference = 22.2).

Histological evaluation of AAA tissues revealed marked structural degradation compared to healthy aortas. H&E staining demonstrated extensive inflammatory infiltration and medial disruption. EVG staining showed fragmentation and loss of elastic lamellae, while Masson’s trichrome staining revealed enhanced collagen deposition. IHC analysis further demonstrated pronounced upregulation of HO-1 in aneurysmal lesions, particularly localized in macrophage-rich regions. The upregulation of HO-1 by heme is accompanied by the induction of H-ferritin, known to also exhibit antioxidant and anti-inflammatory functions. This pattern may suggest a compensatory response to heme stress in the artery wall (Figure 6).

In addition, we performed IHC for IL1β, and TNFα to assess inflammatory activation within AAA tissues (Figure 6). These markers exhibited strong positive staining in aneurysmal lesions, whereas healthy aortas showed minimal or no detectable expression. Magnified views confirmed that H-ferritin was primarily localized in macrophages, reflecting iron sequestration, while IL1β and TNFα were expressed in both macrophages and infiltrating neutrophils, indicating active inflammatory signaling in the AAA wall. Collectively, these data highlight coordinated alterations in iron metabolism and inflammatory pathways in AAA lesions, supporting a mechanistic link between heme-driven oxidative stress, immune cell activation, and vascular wall remodeling.

### 3.6. Heme Induces Inflammatory Responses in Endothelial and Smooth Muscle Cells That Are Restrained by Heme Oxygenase-1

**Figure 7:**
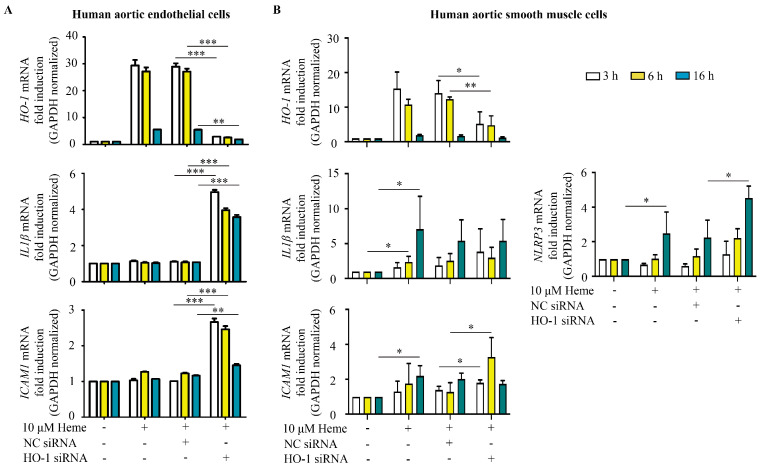
Heme provokes proinflammatory response in human aortic endothelium and smooth muscle cells controlled by heme oxygenase-1. *HO-1* expression was knocked down with small interfering RNAs, then ECs (**A**) and SMCs (**B**) were exposed to heme (10 μM) for 2 h in serum- and antibiotics-free medium. Then, the medium was changed to culture medium with 10% FCS and antibiotics. Relative expression of *HO-1*, *IL1β*, *ICAM1*, and *NLRP3* mRNAs was analyzed 3, 6, and 16 h after the heme treatment. Results are shown as average value ± SEM (n = 3). Data were analyzed by two-way ANOVA with Sidak’s multiple comparisons test. * *p* < 0.05, ** *p* < 0.01, *** *p* < 0.001.

Evidence suggests that endothelial dysfunction represents an early pathological event in AAA development, contributing to elevated inflammation within the degenerating arterial wall [17]. Given that elevated IL1β and ICAM1 are implicated in AAA development [25,26,27,28] and ICAM1 is regulated by IL1β, we analyzed whether knocking down *HO-1* in ECs affects *IL1β* and *ICAM1* expression in response to heme. We found that heme (10 μM) significantly induced *HO-1* expression in ECs (Figure 7A) but did not alter *IL1β* and *ICAM1* expression. Importantly, after knocking down *HO-1*, heme significantly upregulated both *IL1β* and *ICAM1* in endothelium. These results suggest that HO-1 plays a protective role in limiting *IL1β* and *ICAM1* expression during endothelial activation in response to heme.

In contrast to endothelium, SMCs responded to heme exposure by upregulating both *IL1β* and *ICAM1*, but silencing *HO-1* failed to alter *IL1β* mRNA level. Importantly, *NLRP3* induction, a key inflammasome component, occurred after heme treatment, and the induction was even more pronounced after silencing *HO-1.* Accordingly, *ICAM1* was upregulated in SMCs exposed to heme (Figure 7B), and ICAM1 mRNA was further elevated as *HO-1* was knocked down. These findings indicate that heme exerts a broad inflammatory response in vascular resident cells and such responses are under the control of *HO-1*.

### 3.7. Normosang Alleviates the Progression of Abdominal Aortic Aneurysm in Mice

**Figure 8:**
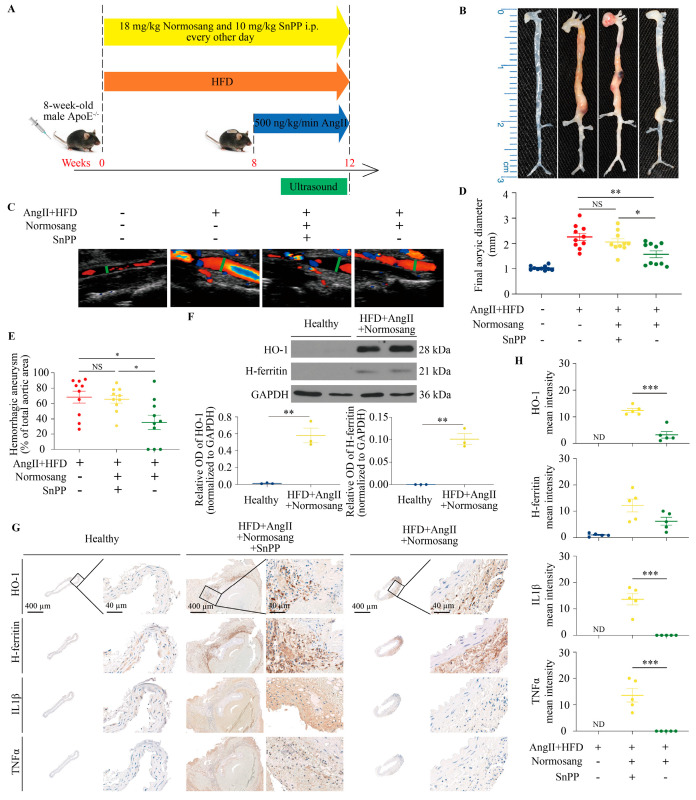
Normosang (heme arginate) retards the progression of angiotensin II-induced abdominal aortic aneurysm in mice via heme oxygenase-1. (**A**), Schematic representation of the experimental design showing intraperitoneal administration of the heme synthesis inhibitor Normosang and the heme degradation inhibitor SnPP in AngII+HFD-treated mice. (**B**), Representative macroscopic images of aortas from four experimental groups: Control, AngII+HFD (AAA), AngII+HFD+Normosang+SnPP, and AngII+HFD+Normosang. (**C**), Representative high-frequency ultrasound frames of aortas. (**D**), Quantification of the final aortic diameters across the four experimental groups. Maximal suprarenal abdominal aortic diameter (in mm) measured from the isolated aorta. (**E**), Quantitative comparison of the percentage of hemorrhagic aneurysms among the AngII+HFD, AngII+HFD+Normosang+SnPP, and AngII+HFD+Normosang groups. Results are shown as average value ± SEM (n = 10). Statistical significance was determined using one-way ANOVA followed by Bonferroni post hoc tests: * *p* < 0.05, ** *p* < 0.01, NS no significant. (**F**), Western blot analysis of HO-1 and H-ferritin in healthy control and AngII+HFD+Normosang-treated mice. HO-1, H-ferritin, and GAPDH were sequentially detected on the same membrane (strip-and-reprobe method). Western blot densitometry values are shown as mean ± SEM (n = 3 per group). ** *p* < 0.01 using unpaired *t*-test. (**G**,**H**), IHC analysis of HO-1, H-ferritin, IL1β, and TNFα in healthy control, AngII+HFD+Normosang+SnPP-treated, and AngII+HFD+Normosang-treated mice. Results are shown as average value ± SEM (n = 5). Statistical significance was determined using one-way ANOVA followed by Bonferroni post hoc tests. Effect size analysis showed large to extremely large effects for all markers (HO-1 effect size = 4.02, mean difference = 9.2; H-ferritin effect size = 1.31, mean difference = 6; IL1β effect size = 4.07, mean difference = 13.6; TNFα effect size = 3.38, mean difference = 13.6). *** *p* < 0.001, ND, not detectable.

To further evaluate the functional role of HO-1 in maintaining vascular integrity and bearing adaptive response during the development of AAA, we treated mice with Normosang (heme arginate) intraperitoneally to upregulate HO-1, with or without SnPP as an inhibitor of HO-1 enzyme activity (Figure 8A). Macroscopic aorta images demonstrated that Normosang administration markedly reduced aneurysm formation and intramural hemorrhage compared to the AngII+HFD group (Figure 8B). Conversely, SnPP co-treatment abolished the protective effect of Normosang, resulting in aneurysm formation similar to the AngII+HFD group.

High-frequency ultrasound imaging confirmed these observations, showing smaller luminal diameters in Normosang-treated mice, whereas SnPP restored aneurysmal expansion (Figure 8C). Final aortic diameters were also significantly reduced by Normosang and partially or fully restored by SnPP (Figure 8D). Quantitative analysis indicated that the percentage of hemorrhagic aneurysms was significantly lower in the AngII+HFD+Normosang group but increased upon SnPP co-treatment (Figure 8E).

Western blot analysis demonstrated that Normosang treatment increased vascular expression of HO-1 and H-ferritin compared to healthy controls (Figure 8F). Accordingly, IHC analysis further confirmed upregulation of HO-1 and H-ferritin in Normosang-treated mice (Figure 8G), supporting the protective role of HO-1 induction in attenuating aneurysm progression. Importantly, expression of pro-inflammatory cytokines IL1β and TNFα was significantly reduced in Normosang-treated aortas (Figure 8H), and such benefit was halted by SnPP, the inhibitor of HO-1 activity. Collectively, these results indicate that induction of HO-1 by Normosang confers vascular protection and suppresses AAA progression, whereas inhibition of HO-1 activity with SnPP abrogates these benefits and exacerbates aneurysm severity.

## 4. Discussion

Pathogenic involvement of vessel wall inflammation has been established in the development of AAA [1,2,3,4,5]. Correlation analyses revealed a significant positive association between the level of inflammatory markers in patients serum (CRP, IL-6) and the expression of *HO-1* mRNA in abdominal aortic wall linking local inflammatory activation with the induction of HO-1 known to be inducible by heme (Figure 2). The observation of the accumulation of heme released from Hb after its oxidation to metHb in human AAA tissue and in AngII-induced AAA of ApoE^−/−^ mice raises the question whether heme is involved in the pathophysiology and HO-1 functions as an adaptive response to control vascular remodeling.

HO-1 (encoded by the *Hmox1* gene), as an inducible stress protein in different organs and tissues, exhibits protective functions at various disease states [10,29,30,31,32]. The main role of HO-1 is to degrade heme and generate carbon monoxide (CO) and biliverdin while simultaneously releasing iron, which is stored within the iron-binding protein ferritin with antioxidant and anti-inflammatory properties [33]. Biliverdin is converted to bilirubin by the cytosolic biliverdin reductase [34,35]. These products exert signaling and cytoprotective activities that alleviate inflammation, regulate vasomotor tone, prevent various cellular injuries, inhibit apoptosis, and provide antioxidant and immunomodulatory functions [33]. Biliverdin and bilirubin were revealed to efficiently scavenge reactive oxygen species [36] and act as modulators of cell signaling [35]. CO was found to mitigate vascular- and immuno-related dysfunctions [37,38]. In addition to HO-1 derived products, the essential function of this enzyme is to counteract oxidative tissue injury triggered by free heme [39]. It potentiates cellular damage mediated by leukocytes and reactive oxygen [40], and mediates the oxidation of low density lipoprotein and endothelial injury [41]. Heme cytotoxicity was shown to be the consequence of endoplasmic reticulum stress in atherosclerotic plaque progression controlled by HO-1 [23]. Heme as an extracellular signaling molecule was also demonstrated to affect the innate immune response through a receptor-mediated mechanism in macrophages [42] and provoke NF-κB activation of endothelium [43]. These alarm signals are particularly important when there is cellular damage and cells suddenly release large amounts of heme, which is likely amplified in injured tissues containing high amounts of heme such as the AAA [20]. These observations provide a broad spectrum of fundamental mechanisms by which HO-1 catabolizes heme and subsequently protects tissues under conditions of various cellular insults including heme-stress.

The first studies that demonstrated the paramount role of HO-1 in maintaining vascular integrity in AAA and suppressing the pathogenesis of AAA were carried out in HO-1-deficient mice [6,7]. The complete loss of HO-1 was found to increase AAA incidence and rupture rate, and drastically increase aneurysmal area and severity in AngII-induced AAA formation in apolipoprotein-deficient mice [6]. Furthermore, patients with AAA are less frequently carriers of short (<25 GT) repeats, associated with an increased HO-1 upregulation in response to inflammatory stimuli in the HO-1 gene promoter, than healthy subjects [8]. Therefore, the increased HO-1 activity has been offered to be an attractive therapeutic approach. Subsequently, several HO-1 inducers have been identified and tested in animal models for preventing or mitigating AAA [44,45,46,47,48].

Expression of HO-1 is regulated by heme [12,13,14], and it was used in acute life-threatening porphyric attacks with the high risk of harmful effects on the vasculature, such as vasculitis, thrombosis, and disseminated intravascular coagulation [49,50,51,52,53]. In order to avoid vascular damage resulted from heme, heme arginate (Normosang) treatment was introduced for acute porphyric attacks without evidence of vascular side effects [15,54]. Heme arginate is an effective *HO-1* gene regulator, but unlike heme, it does not amplify oxidative cellular damage mediated by leukocytes and does not provoke oxidative modification of low-density lipoprotein [16].

Drawing upon these lines we performed an experiment employing AngII-provoked AAA in ApoE^−/−^ mice to seek the signatures of heme stress in such pathology. RNA-seq analysis revealed that aneurysmal tissues are characterized by robust transcriptional activation of heme metabolism-related genes, including *Hmox1*, accompanied by marked upregulation of inflammatory mediators such as *IL1β* and *IL16*. These findings support a model in which heme exposure and oxidative stress jointly promote inflammatory and degenerative remodeling during AAA development. Histological and biochemical analyses confirmed increased Hb and heme accumulation in murine AAA lesions, consistent with intramural hemorrhage and erythrocyte degradation as key pathogenic features. Elevated HO-1 expression in aneurysmal regions likely represents a compensatory defense against heme-mediated oxidative injury and maladaptive cellular activation. However, excessive or dysregulated heme turnover may also exacerbate oxidative stress, emphasizing the delicate balance of heme metabolism in vascular homeostasis.

Intervention experiments further support the causal role of heme in AAA progression. Upregulation of HO-1 by heme arginate significantly attenuated aneurysm formation and reduced intramural hemorrhage. Conversely, inhibition of HO-1 activity by SnPP abolished these protective effects, resulting in aggravated inflammation, aneurysmal dilation, and rupture. Inhibition of HO-1 activity by SnPP likely impairs the generation of cytoprotective products of heme catabolism and the detoxification of pro-oxidant and proinflammatory heme. Collectively, these results highlight that the heme-HO-1 axis is a pivotal modulator of AAA pathogenesis.

Normosang treatment of mice enhanced the expression of H-ferritin as well within aortic wall, a post-transcriptionally regulated protein by iron [55], previously found to protect cells against oxidative insults and retard inflammation [56]. Surprisingly, SnPP reduced but did not prevent the induction of H-ferritin by Normosang. This may reflect a direct translational regulation of ferritin by heme [57] independently from iron released from heme by HO-1, allowing cooperative adaptive actions against vascular remodeling.

SMCs upregulate *IL1β* and *ICAM1,* known to be regulated by IL1β in response to heme exposure, and the key inflammasome component, *NLRP3,* is also induced by such insult. Importantly, silencing of *HO-1* is required to provoke inflammatory response by heme in aortic ECs as reflected by *IL1β* and *ICAM1* induction. These findings also support the protective function of HO-1 in controlling the inflammation triggered by heme in AAA.

The accumulation of Hb and heme in AAA and the oxidation of heme-iron to ferri state leading to the formation of metHb and liberation of heme represent a damaging signal within the aortic wall. The elevation of circulating inflammatory markers, such as CRP and IL-6, may result from local vascular inflammation provoked by heme. The signatures of rise an adaptive response, namely HO-1 and H-ferritin inducible by heme also reflect such an insult. Protection of HO-1 induction in aortic wall against AAA provides not only a therapeutic advance but also indicates the pathophysiologic role of heme in AAA, enlightening a novel direction for AAA treatment.

Despite providing important mechanistic insights, several limitations should be acknowledged. First, systemic hematologic parameters (hematocrit and Hb levels) were not measured in this study. Heme arginate is known to inhibit endogenous heme synthesis, and thus changes in systemic heme availability could theoretically influence aneurysm progression independently of HO-1 induction. Importantly, however, the protective effects of heme arginate were abolished by HO-1 inhibition with SnPP, supporting a primary role for HO-1-dependent pathways relevant to aneurysm pathogenesis, including regulation of vascular inflammation, oxidative stress, and SMCs survival. The number of human AAA tissue samples was limited, and in vitro experiments in ECs and SMCs cannot fully recapitulate the complex aortic environment. Likewise, the murine AngII-induced AAA model does not entirely reflect chronic human disease, and long-term effects of HO-1 modulation remain to be investigated. These limitations highlight opportunities for future studies in larger human cohorts and advanced experimental models.

## Figures and Tables

**Table 1:** Differential expression of heme metabolism-related genes in abdominal aortic aneurysm versus control aortas in mice.

Gene	Log_2_FC	FDR	Regulation	Function
*Tfr2*	3.86	0.003	↑	Transferrin receptor, iron uptake
*Hmox1*	1.71	0.105	↑	Heme degradation
*Blvrb*	0.41	0.082	↑	Biliverdin reductase B
*Slc25a37*	0.34	0.12	↑	Mitochondrial iron importer
*Slc40a1*	5.65	0.0015	↑	Ferroportin 1, cellular iron export
*Lcn2*	−2.15	0.0009	↓	Iron-binding protein, inflammation
*Fth1*	−0.72	0.02	↓	Ferritin heavy chain, iron storage
*Tfrc*	−1.28	0.054	↓	Transferrin receptor 1, cellular iron uptake
*Slc11a2*	−0.35	0.12	↓	Divalent metal transporter 1, endosomal iron importer

Values are log_2_ fold changes (mouse AAA vs. control) from RNA-seq analysis. Upregulated genes (↑) and downregulated genes (↓) were identified based on adjusted *p* < 0.05. FDR, false discovery rate.

## Data Availability

The RNA-seq data under this study are available in the NCBI BioProject database under accession number PRJNA1196652 and PRJNA1358949.

## Abbreviations

AAA	Abdominal aortic aneurysm
AngII	Angiotensin II
ApoE^−/−^	Apolipoprotein E-deficient
a.u.	Arbitrary units
CO	Carbon monoxide
CRP	C-reactive protein
CTA	Computed tomography angiography
EVG	Verhoeff-Van Gieson
FBS	Fetal bovine serum
HAoECs	Human aortic endothelial cells
HAoSMCs	Human aortic smooth muscle cells
Hb	Hemoglobin
H&E	Hematoxylin-eosin
HFD	High-fat diet
HO-1/Hmox1	Heme oxygenase-1
ICAM1	Intercellular adhesion molecule 1
IHC	Immunohistochemistry
IL1β	Interleukin-1 beta
IL-6	Interleukin-6
metHb	Methemoglobin
NLRP3	NOD-, LRR- and pyrin domain-containing protein 3
OxyHb	Oxyhemoglobin
PCA	Principal component analysis
qRT-PCR	Quantitative reverse transcription-polymerase chain reaction
RNA-seq	RNA sequencing
SEM	Standard error of the mean
siRNA	Small interfering RNA
SnPP	Tin protoporphyrin IX
TBST	TBS-Tween
